# Increased mucosal IL-12 expression is associated with relapse of ulcerative colitis

**DOI:** 10.1186/s12876-021-01709-5

**Published:** 2021-03-17

**Authors:** Kazuhiko Uchiyama, Tomohisa Takagi, Katsura Mizushima, Mariko Kajiwara-Kubota, Saori Kashiwagi, Yuki Toyokawa, Makoto Tanaka, Yuma Hotta, Kazuhiro Kamada, Takeshi Ishikawa, Hideyuki Konishi, Mitsuo Kishimoto, Yuji Naito, Yoshito Itoh

**Affiliations:** 1 Department of Endoscopy and Ultrasound Medicine, Molecular Gastroenterology and Hepatology, Kyoto Prefectural University of Medicine, 465 Kajiicho Hirokoji Kawaramachi Kamigyo-ku, Kyoto, 602-8566 Japan; 2 Department for Medical Innovation and Translational Medical Science, Graduate School of Medical Science, Kyoto Prefectural University of Medicine, Kyoto, 602-8566 Japan; 3 Department of Surgical Pathology, Kyoto City Hospital, Kyoto, 604-8845 Japan; 4 Department of Endoscopy and Ultrasound Medicine, Kyoto Prefectural University of Medicine, Kyoto, 602-8566 Japan

**Keywords:** IL-12, IL-23, Ulcerative colitis, Rectal mucosa, Relapse

## Abstract

**Background:**

The role of IL-12/23 in the pathogenesis of ulcerative colitis (UC) is unclear. We analyzed mucosal IL-12/23 expression and its relationship with endoscopic severity, histological activity, and UC relapse.

**Methods:**

Rectal biopsies were collected from 70 UC patients with clinical remission. IL-12, IL-23, IFN-γ, IL-17A, and IL-17F mRNA expression was measured by real-time PCR. Endoscopic severity and histological activity were evaluated using the Mayo endoscopic subscore (MES) and the Geboes score, respectively.

**Results:**

The longest follow-up period was 51 months. Thirty-four patients relapsed during the study period. Samples from these subsequently relapsed patients formed the “relapse” group, while those from patients that did not relapse formed the “remission” group. IL-12 (*P* = 0.0003) and IL-23 (*P* = 0.014) mRNA expression was significantly higher in the relapse than the remission group. Expression of IL-23 (*P* = 0.015) but not IL-12 (*P* = 0.374) was correlated with MES. However, in patients with an MES of 0 and 1, IL-12 expression was statistically higher in the relapse than the remission group (*P* = 0.0015, *P* = 0.0342). IL-12 and IL-23 expression did not vary significantly between histologically active and inactive mucosa; both were higher in histologically inactive patients in the remission group (IL-12: *P* = 0.0002, IL-23: *P* = 0.046).

**Conclusions:**

Rectal IL-12 and IL-23 expression was elevated in the relapse group, but IL-12 was more strongly associated with UC relapse, irrespective of endoscopic severity and histological activity. Mucosal IL-12 was elevated in patients with deep mucosal healing. Our results suggest an important role of IL-12 in UC pathogenesis and the molecular mechanism of UC relapse.

**Supplementary Information:**

The online version contains supplementary material available at (10.1186/s12876-021-01709-5)

## Background

Inflammatory bowel diseases (IBD), which include ulcerative colitis (UC) and Crohn’s disease (CD), are chronic conditions characterized by recurrent intestinal inflammation. A complex network of immune cells, such as T-helper cells, and cytokines such as interleukins, are involved in the pathogenesis of IBD. Since inflammatory mediators have been implicated in the pathogenesis of IBD, these molecules have been utilized as therapeutic targets for its treatment. TNF-α was the first molecule to be identified as a therapeutic target to control IBD [1]. Many TNF-α inhibitors have been clinically approved for treatment of CD and UC. Recently, refractory or loss of response to TNF-α inhibitors [2] and adverse events associated with anti-TNF-α therapy[3] have been observed in patients with IBD. Therefore, other cytokines have been targeted to developed new therapeutics and have been clinically applied to treat IBD. The IL-12/23 axis is among the fundamental pathways targeted by these new therapies to treat intestinal inflammation [4].

IL-12 was first identified as a natural killer cell stimulatory factor (NKSF) promoting interferon (IFN)-γ production [5]. The IL-12 cytokine family contains four cytokines, IL-12, IL-23, IL-27, and IL-35, which contain a helical α-subunit (p35, p19, and p28) and a β-subunit (p40 and EBI3) [6, 7]. IL-12 contains the p35 and p40 domains, IL-23 contains p19 and p40, IL-27 contains p28 and EBI3, and IL-35 contains p35 and EBI3. The IL-12 receptor is also heterodimeric protein consisting IL-12Rβ1 and IL-12Rβ2 domains. IL-12 binds to the IL-12 receptor and activates Janus kinase-2 (JAK-2) and tyrosine kinase-2 (TYK-2), resulting the activation of signal transducer and activator of transcription-4 (STAT-4). This is the primary pathway for IFN-γ induction and Th1 differentiation [8, 6]. IL-12 is secreted by monocytes, macrophages, and dendritic cells upon binding of pathogenic structures to toll-like receptors, and it drives the differentiation of naïve T cells into IFN-γ producing Th1 cells [9]. IL-12 thus connects innate and adaptive immunity [10]. IL-23, a member of the IL-12 cytokine family, is also predominantly produced by activated dendritic cells, and plays an important role in amplifying Th17 proliferation resulting in IL-17A and IL-17F secretion.

The monoclonal antibody ustekinumab targets the p40 subunit of IL-12 and IL-23, and has been approved for the treatment of CD [11] and UC [12]. The phase 3 trial of ustekinumab as an induction and maintenance therapy for UC demonstrated that ustekinumab was more effective than placebo for inducing and maintaining remission in patients with UC [12]. Recently, a real-world cohort study reported a similar efficacy of ustekinumab in treating UC as the phase 3 ustekinumab clinical trial [13]. It has also been reported that IL-12 mRNA expression is significantly elevated in the inflamed mucosa of UC patients who underwent colorectal surgery [14]. Recently, Chapuy et al. reported that CD163^−^ monocyte-like cells increased the amount of IL-8^+^IL-17^±^IFNγ^±^T cells via expression of IL-1β and IL-12 in the inflamed mucosa of UC patients [15]. They also reported that IL-23 levels are elevated in CD163^−^ monocyte-like cells with inflamed mucosa in UC patients. Based on results of clinical trials and data using clinical materials, IL-12 and IL-23 are considered essential in the pathogenesis of UC. However, the relationship between long-term prognosis of UC and mucosal IL-12 or IL-23 expression has not been investigated.

In the present study, we investigated the association between mucosal IL-12 expression and clinical outcomes of UC patients, including endoscopic findings and histological examination. Additionally, we have also investigated mucosal expression of IL-23, which is a member of the IL-12 family and therapeutic target of ustekinumab. Furthermore, we compared mucosal IL-23 expression with IL-12 expression.

## Methods

### Participants

A total of 70 patients with ulcerative colitis were enrolled in this study. All patients attended the gastroenterology outpatient clinic at the Hospital of the Kyoto Prefectural University of Medicine and were diagnosed with clinical remission. The characteristics of the enrolled patients are shown in Table 1.

**Table 1:** Patient characteristics and background

Total number	70
Sex (female/male)	32/38
Age (years)	45.0 ± 16.0
Disease duration (month)	111.2 ± 104.0
Smoking history (%)	7 (10.0)
Disease location (%)	
Extensive	54 (77.1)
Left-sided	11 (15.7)
Rectum	5 (7.1)
Current medication (%)	
5-Aminosalicylates	62 (88.6)
Prednisolone	1 (1.4)
Azathioprine	13 (18.6)
Biologics	
IFX	3 (4.3)
ADA	5 (7.1)
GLM	2 (2.9)

### Diagnostic evaluation

Three endoscopists who were blinded to the patient details of the endoscopic images and clinical data evaluated all endoscopic images. Two were expert endoscopists (experts A and B) who had previously performed > 8000 conventional colonoscopies, while the other was a non-expert (non-expert C) who had previously performed < 1000 conventional colonoscopies. This analysis was performed based on the previous report [16].

### Assessment of disease activity

Clinical disease activity was determined using the Lichtiger Colitis Activity Index (LCAI) [17]. All patients enrolled in this study were in clinical remission, defined as a score of 4 or below on LCAI. The relapse of ulcerative colitis was defined as aggravation of clinical symptoms of ulcerative colitis, and is characterized by an aggravation of endoscopic findings.

### Sample collection

All participants underwent total colonoscopy, and endoscopic activity was determined using the Mayo endoscopic subscore (MES). Rectal biopsy was performed to analyze cytokine mRNA expression and histological activity, which is the same diagnostic area as the total colonic mucosal diagnosis evaluated by MES. All specimens were collected during the remission phase. Based on whether the patient later relapsed or not, the samples were grouped into “relapse” and “remission”, respectively.

### Histopathological assessment

Inflammation in the biopsy specimen was evaluated according to the Geboes score [18] by an expert pathologist. Biopsies were collected from the same sites that were subjected to MES, and active histological inflammation was defined as a Geboes score ≥ 2B.1.

### mRNA analysis

The mRNA expression of human colonic mucosal cytokines was determined by real-time polymerase chain reaction (RT-PCR) using biopsy samples from patients with UC. A total of 70 samples from all patients were analyzed. Total RNA was isolated from human biopsy samples by the acid guanidinium phenol-chloroform method, using an ISOGEN kit (Nippon Gene, Tokyo, Japan). The concentration of RNA was determined by the ratio of absorbance at 260 and 280 nm (A260/280). The isolated RNA was stored at − 80 °C until RT-PCR was performed. Extracted RNA (1 μg) was reverse-transcribed into cDNA at 42 °C for 40 min, using 100 U/mL of reverse transcriptase (Takara Biomedicals, Shiga, Japan) and 0.1 μM of oligo(dT)-adapter primer (Takara Biomedicals) in a 50-μL reaction mixture. Real-time PCR for human and mouse Serpin B1 was carried out with the 7300 Real-time PCR DNA-binding dye SYBR green I for the detection of PCR products. The reaction mixture (RT-PCR kit, Code RRO43A; Takara Biochemicals) contained 12.5 μL Premix Ex Taq, 2.5 μL SYBR green I, custom-synthesized primers, ROX reference dye, and cDNA (equivalent to 20 ng total RNA) in a final reaction volume of 25 μL. The PCR settings were as follows: the initial denaturation for 15 s at 95 °C was followed by 40 cycles of amplification for 3 s at 95 °C and 31 s at 60 °C, with subsequent melting curve analysis increasing the temperature from 60 to 95 °C. A total of 22 cytokine mRNAs (IFNγ, IL-12, IL-17A, IL-17F, IL-23) were quantified from biopsy specimens, and the sequences of primers are shown in Additional file 1: Fig. S1. GAPDH was used as an internal control. This analysis was performed based on the previous report [19].

### Statistical analysis

Continuous data were described as mean ± standard deviation (SD), if normally distributed, or median and interquartile range [IQR] (25%, 75%), if not normally distributed. Kappa values of < 0.20, 0.21–0.40, 0.41–0.60, 0.61–0.80, and > 0.80 indicate poor, fair, moderate, good, and excellent agreement, respectively. Values of *p* < 0.05 were considered statistically significant. The analysis of variance (ANOVA) was performed to assess the trend of the mean, stratified according to the normally distributed continuous variables; the trend test was based on liner contrast. If a non-parametric method was required, the Jonckheere–Terpstra trend test was performed. Values of *p* < 0.05 were considered statistically significant. All analyses were performed with SPSS version 22.0 (IBM Japan, Ltd, Japan). The non-relapse rate was plotted using the Kaplan–Meier method and compared using the log-rank test. Thirty months was defined as the time interval between endoscopic diagnosis and relapse (censored observation). This analysis was performed based on the previous report [16].

## Results

### Patient baseline demographic variables

**Fig. 1:**
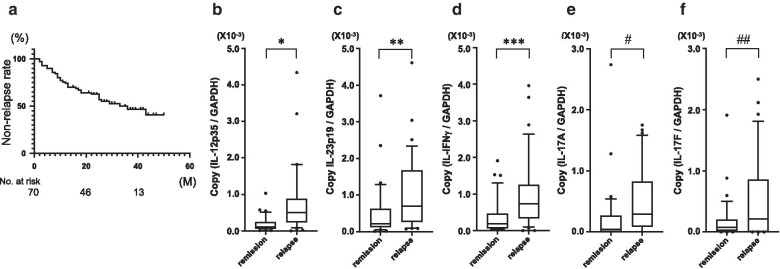
The non-relapse rate for all UC patients enrolled in this study (**a**). The comparison of colonic mucosal IL-12 mRNA expression (**b**), IL-23 mRNA expression (**c**), IFN-γ mRNA expression (**d**), IL-17A expression (**e**), and IL-17F expression (**f**) between the patients exhibiting remission and relapse (remission: n = 36, relapse: n = 34). *P = 0.0003, **P = 0.014, ***P = 0.0014, ^#^ *P* = 0.0277, ^##^ *P* = 0.0125

The characteristics of the 70 patients who were enrolled in this study are listed in Table 1. Thirty-four patients (48.6%) showed relapse during study period, and the median follow-up time of patients in remission group was 31.0 months (15–51) and in relapse group was 11.5 months (2–43) (Fig. 1a). In this study, we determined the interval of follow-up period from the point of the diagnostic endoscopy, and the longest follow-up period was 51 months for patients in the remission group.

### Validation of endoscopic findings

The kappa values for interobserver variability across the three endoscopists in diagnosing MES were excellent. The kappa value for the diagnosis of MES for the expert (A) and expert (B) was 0.823. The kappa values for interobserver variability between the non-expert (C) with expert (A) and (B) were 0.889 and 0.822, respectively.

### Cytokine mRNA expression compared to relapse and remission

IL-12 and IL-23 mRNA expression in patients with remission and relapse are shown in Fig. 1b, c. Mucosal IL-12 and IL-23 mRNA expression was significantly elevated in the relapse group compared to the remission group (*P* = 0.0003, Fig. 1b for IL-12: *P* = 0.014; Fig. 1c for IL-23). IFN-γ is produced by Th1 cells and promotes the differentiation by IL-12. IFN-γ mRNA expression was significantly higher in the relapse group than in the remission group (Fig. 1d; *P* = 0.0014). IL-17A and IL-17F are produced by Th17 cells which is promoted to be differentiated by IL-23. The mRNA expression of IL-17A and IL-17F was significantly higher in the patients of relapse group as compared to remission group (Fig. 1e; IL-17A: *P* = 0.0277, Fig. 1f; IL-17F: *P* = 0.0125).

### Endoscopic evaluation and IL-12/23 expression

**Fig. 2:**
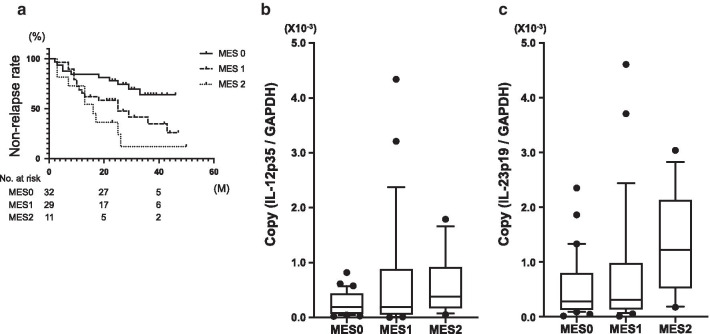
The non-relapse rate for patients with UC according to MES (**a**). Log-rank test: *P* = 0.0332. The distribution of IL-12 mRNA expression in each MES (**b**). *P* = 0.374 for trend: ANOVA linear contrast test. The distribution of IL-23 mRNA expression in each MES (**c**). *P* = 0.015 for trend: ANOVA linear contrast test. (MES 0: n = 30, MES 1: n = 27, MES 2: n = 13)

**Fig. 3:**
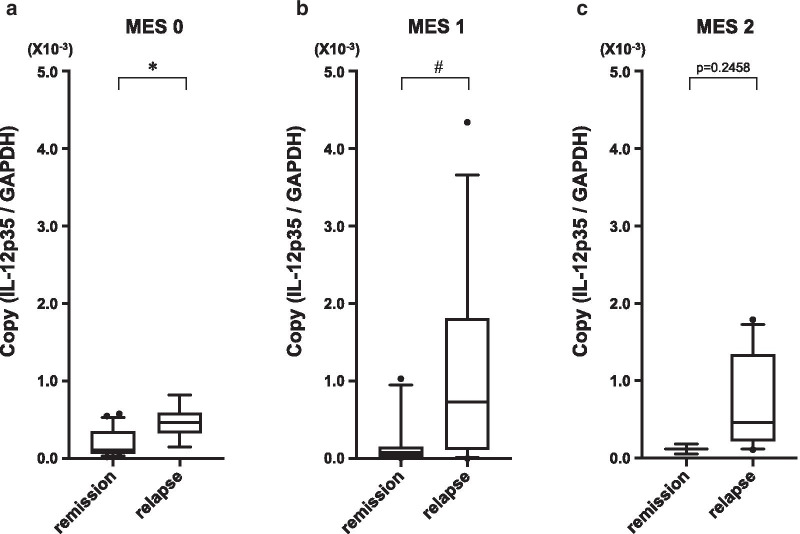
The comparison of colonic mucosal IL-12 mRNA expression in patients with an MES of 0 (**a**). (remission: n = 22, relapse: n = 8), MES of 1 (**b**) (remission: n = 12, relapse: n = 15), and MES of 2 (**c**) (remission: n = 2, relapse: n = 11) between the patients exhibiting remission and relapse. *P = 0.0015, # *P* = 0.0342

**Fig. 4:**
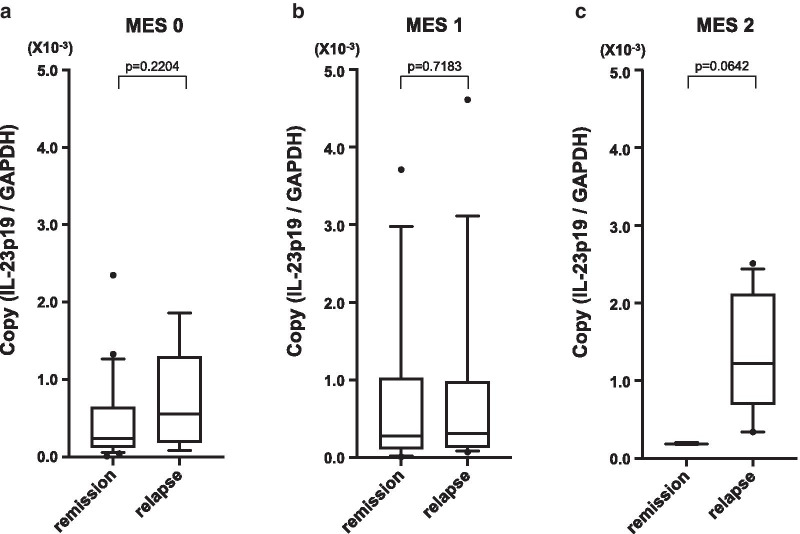
The comparison of colonic mucosal IL-23 mRNA expression in patients with an MES of 0 (**a**) (remission: n = 22, relapse: n = 8), MES of 1 (**b**) (remission: n = 12, relapse: n = 15), and MES of 2 (**c**) (remission: n = 2, relapse: n = 11) between the patients exhibiting remission and relapse

The relapse rate during the study period for each endoscopic classification was 26.7% (8/30) for MES 0, 55.6% (15/27) for MES 1, and 84.6% (11/13) for MES 2. These relapse rates were statistically correlated with endoscopic findings using the log-rank test (Fig. 2a). IL-12 and IL-23 mRNA expression for each endoscopic classification are shown in Fig. 2b, C. IL-12 mRNA expression did not correlate with endoscopic severity diagnosed by MES (Fig. 2b; *P* = 0.374), but IL-23 mRNA expression increased with MES2, and was correlated with endoscopic severity (Fig. 2c; *P* = 0.015). We then investigated the relationship between IL-12 mRNA expression and clinical relapse in each endoscopic severity class and found that IL-12 mRNA was significantly increased in relapse cases in MES0 and MES1 (Fig. 3a–c). In contrast, IL-23 mRNA expression was not different between the relapsed group and the remission group according to the endoscopic severity (Fig. 4a–c).

### Histological evaluation and IL-12/23 expression

**Fig. 5:**
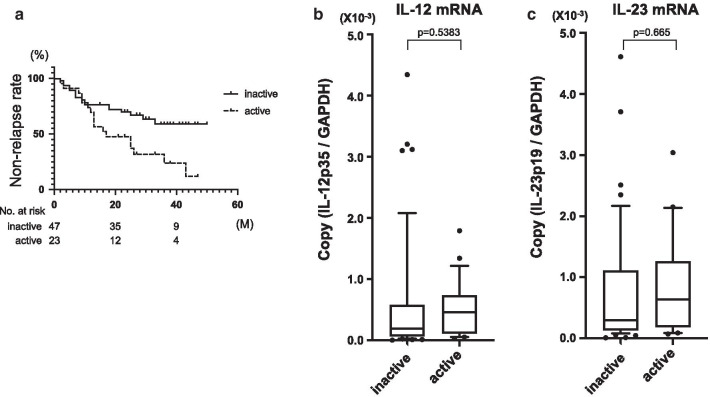
The non-relapse rate for UC patients according to histological activity (**a**). Log-rank test: *P* = 0.009. (**b**) The distribution of IL-12 mRNA expression in histologically active and inactive groups. (**c**) The distribution of IL-23 mRNA expression in histologically active and inactive groups (inactive: n = 47, active: n = 23)

**Fig. 6:**
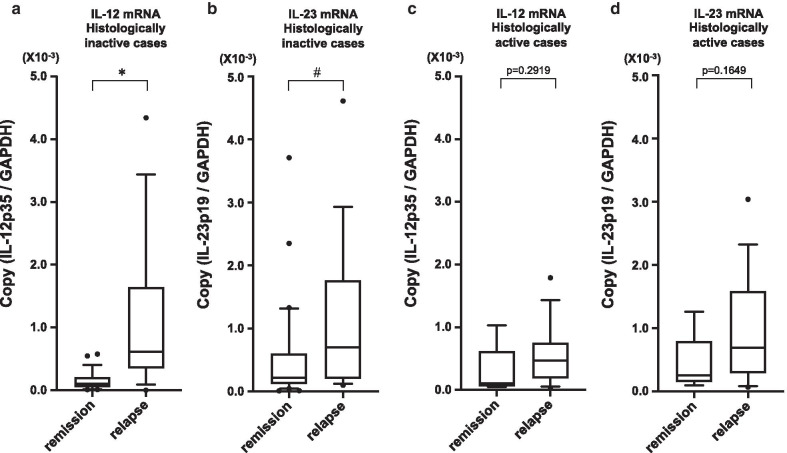
The comparison of colonic mucosal IL-12 (**a**) and IL-23 (**b**) mRNA expression in histologically inactive patients (remission: n = 30, relapse: n = 17). The comparison of colonic mucosal IL-12 (**c**) and IL-23 (**d**) mRNA expression in histologically active patients (remission: n = 6, relapse: n = 17). *P = 0.0002, #*P* = 0.046

The relapse rate during the study period upon histological diagnosis was 36.2% (17/47) for histologically inactive samples, 73.9% (17/23) for histologically active samples. These relapse rates were statistically correlated with histological activity using the log-rank test (Fig. 5a). There was no difference in IL-12 and IL-23 mRNA expression between histologically inactive and active samples (Fig. 5b; IL-12:*P* = 0.5383, Fig. 5c; IL-23:*P* = 0.665). Among the patients exhibiting histological inactivity, IL-12 mRNA expression was significantly higher in the relapse group than in the remission group (*P* = 0.0002) (Fig. 6a). IL-23 mRNA expression was also elevated in the relapse group in the patients exhibiting histological inactivity (*P* = 0.046), but the elevation of IL-12 expression was more marked than that of IL-23 (Fig. 6b). In contrast, there were no differences in both IL-12 and IL-23 mRNA expression between remission and relapse patients exhibiting histological activity (Fig. 6c, d).

## Discussion

In the present study, we demonstrated the role of mucosal IL-12 as a predictor of relapse in patients with UC. Furthermore, as IL-12 expression was not correlated with both endoscopic severity and histological activity, mucosal IL-12 expression was considered as an independent factor in predicting clinical outcome of patients with UC. To our knowledge, this study is the first to correlate mucosal IL-12 expression with long-term clinical outcomes in patients with UC in remission. Our results indicate that IL-12 may play an important role in the pathogenesis of UC, especially associated with long-term clinical outcomes.

Various prognostic factors have been reported for UC. Endoscopic evaluation of colonic mucosa is among the most popular approaches to predict clinical outcome of patients with UC. It has been reported that clinical outcomes, including colectomy with infliximab treatment, are related to endoscopic findings 8 weeks after treatment [20]. In that report, MES 0–1 was defined as mucosal healing, and the patients exhibiting mucosal healing 8 weeks after treatment showed statistically higher colectomy-free rate compared to MES 2–3 through 54 weeks (MES 0: 95%, MES 1: 95%, MES 2: 87%, MES 3: 80%). The concordance between colectomy-free rate and MES has also been demonstrated by Laharie et al. [21]. They reported that patients with an MES of 0–1 before infliximab treatment exhibited statistically higher colectomy-free rate compared to those with an MES of 2–3 until 48 weeks follow up (MES 0: 100%, MES 1: 94%, MES 2: 68%, MES 3: 45%, *P* = 0.02: MES 0–1 vs MES 2–3). Multivariate analysis revealed that endoscopic severity was the most important factor associated with colectomy. Recently, it has been reported that it is important to consider MES 0 and MES 1 separately with regard to clinical outcome of UC patients. Barreiro-de Acosta et al. [22] reported a statistical difference in relapse rates between patients with an MES of 0 and 1. Patients with an MES of 0 showed 19.3% relapse compared to 41.0% with an MES of 1 (*P* < 0.001) at the end of the follow-up period. These reports indicate that endoscopic evaluation of colonic mucosa in patients with UC can predict clinical outcomes and, in our study, UC relapse is also a clinical outcome statistically associated with MES. Several reports concerning the molecular background of endoscopic evaluation and clinical outcomes have been published. It has been reported that mRNA expression of mucosal cytokines, such as IL-33, IL-6, and IL-10, was upregulated in patients with an MES of 1 compared to those with an MES of 0 [23]. Although this study did not investigate the relationship between clinical outcome and mucosal cytokine expression, it has been revealed that colonic mucosa diagnosed as MES 1 had a different cytokine profile than those diagnosed as MES 0. There are few reports demonstrating the relationship between mucosal cytokine expression and clinical outcome in UC patients with clinical remission. Mucosal IL-8 protein levels in the rectum have been reported to be elevated in patients with relapse [24]. More recently, rectal Th/Treg-related mucosal gene expression of IL-17A, IL-17F, and IL-21 were associated with relapse in UC patients [25]. In that report, there was no alteration in the expression of IL-12 and IL-23 between patients with and without relapse. However, these two reports included a small sample size, and the number of patients exhibiting relapse were 16 and 6, respectively. In the present study, 34 and 36 cases were included in the relapse and remission groups, respectively. Therefore, a more comprehensive and accurate analysis of mucosal cytokine expression was possible. Furthermore, our study involved the validation of endoscopic diagnosis of MES by three endoscopists, and histological analysis. These aspects provide novelty to your study. We found increased expression of both IL-12 and IL-23 mRNA in the rectal mucosa the relapse group compared to the remission group. The expression of IFN-γ (which induces production from Th1 cells via IL-12 stimulation), IL-17A and IL-17F, which are produced by Th17 cells by stimulation of with IL-23 stimulation, were also increased in patients with relapse compared to those in remission. However, the degree of upregulation of IL-12 and IFN-γ in the relapse group was more marked than that of IL23, IL-17A, and IL-17F. These results indicate that the IL-12 axis is more relevant to relapse of UC in patients compared to the IL-23 axis. In the present study, similar to previous reports, patients with an MES of 1 exhibited a higher relapse rate compared to those with an MES of 0. IL-12 mRNA expression was significantly higher in the patients in the relapse group than that in the remission group in both the MES 0 and MES 1 groups. This means that mucosal IL-12 expression can help identify patients exhibiting relapse even in patients with deep mucosal healing, defined as MES 0. Interestingly, IL-23 mRNA expression did not differ between the remission and relapse groups for each endoscopic grade. These results indicate that IL-12 mRNA expression is a predictor of relapse independent of the severity of endoscopic findings.

Histological evaluation of inflammation has been recognized as another important approach to predict relapse in patients with UC. Zenlea et al. [26] analyzed clinical factors associated with relapse of UC in patients. According to their report, univariate analysis revealed that MES and Geboes histology grade were significantly associated with subsequent clinical relapse, but only the histology grade remained significant in a multivariate model. It has also been reported that histological remission is more important than endoscopic evaluation in clinical course of corticosteroid use and acute severe colitis requiring hospitalization [27]. In the present study, the patients with histological activity showed a higher rate of relapse compared to patients exhibiting histological inactivity. Although there was no difference in IL-12 and IL-23 mRNA expression between histologically inactive and active patients, IL-12 was significantly elevated in histologically inactive cases of the relapse group. IL-23 was also elevated in histologically inactive cases of the relapse group, but the degree of increased expression of IL-12 was more marked than that of IL-23. These results indicate that IL-12 mRNA expression is a predictor of relapse independent of histological activity.

## Conclusions

In conclusion, upregulation of IL-12 in the rectal mucosa of patients with UC in clinical remission was associated with relapse. IL-12 upregulation is independent of both endoscopic severity and histological activity with regard to UC relapse. Although MES 0 has been defined as deep endoscopic mucosal healing and reported to show a lower relapse rate, mucosal IL-12 expression was statistically elevated in patients exhibiting relapse, even in patients with an MES of 0. Histologically inactive has been recently defined as histological healing and is reported to have a lower relapse rate, but mucosal IL-12 expression was elevated even in patients with histologically inactive UC. These results indicate the important role of IL-12 in the pathogenesis of UC, even in patients with mucosal healing verified by endoscopy and histology. Thus, treatment to block IL-12 activity by monoclonal antibodies such as ustekinumab may be an ideal strategy to prevent relapse of UC in patients.

## Supplementary information

**Additional file 1: Fig. S1** Real-time PCR primer sequences of IL12p35, IL-23p19, IFN-γ, IL-17A, and IL-17F.

## Data Availability

The datasets used and analyzed during the current study available from the corresponding author on reasonable request.

## Abbreviations

UC: Ulcerative colitis
MES: Mayo endoscopic subscore
