# Supplementary material for: Increased mucosal IL-12 expression is associated with relapse of ulcerative colitis
Source: BMC Gastroenterol. 2021 Mar 17;21:122. doi: 10.1186/s12876-021-01709-5 (PMC7968323; doi:10.1186/s12876-021-01709-5)
Supplement: Supplementary file 1 — Additional file 1: Fig. S1 Real-time PCR primer sequences of IL12p35, IL-23p19, IFN-γ, IL-17A, and IL-17F. [file 12876_2021_1709_MOESM1_ESM.pdf]

Supplemental figure 1

	**Forward**	**Reverse**
**IL-12p35**	CCTTCACCACTCCCAAAACCT	TGTCTGGCCTTCTGGAGCAT
**IL-23p19**	CAGCAACCCTGAGTCCCTAA	TCAACATATGCAGGTCCCACT
**IFN-γ**	CCAACGCAAAGCAATACATGA	TTTTCGCTTCCCTGTTTTAGCT
**IL-17A**	GCTGAGCCTGGAGGCCATA	TGGGCATCCTGGATTTCGT
**IL-17F**	GTCCGGAGGAAGCACCAA	TCACCAGCACCTTCTCCAACT
